# Supplementary material for: Are carbon emissions trading and green financial instruments synergistic? -Comprehensive quantitative research based on content analysis
Source: PLoS One. 2024 Mar 7;19(3):e0298601. doi: 10.1371/journal.pone.0298601 (PMC10919601; doi:10.1371/journal.pone.0298601)
Supplement: S1 Data — (ZIP) [file pone.0298601.s001.zip › Data/地级市Z_F工作报告.zip!/─■╧─/╣╠╘¡╩╨/└┤╫╘╣1⁄2╓┌║┼í╛┬φ┐╦╩2╛▌═°í┐_2012.docx]

各位代表：

　　现在，我代表市人民政府向大会作政府工作报告，请予审议，并请市政协委员和列席人员提出意见。

　　2011年工作回顾

　　2011年是实施“十二五”规划的第一年。一年来，在自治区党委、政府和市委的坚强领导下，在市人大和政协的监督支持下，我们团结带领全市各族人民，深入贯彻落实科学发展观，坚持以调结构、促转型、抓项目、夯基础、惠民生、保稳定为目标，奋力推进新型工业化、城镇化、农业产业化，全力抓好事关经济社会发展的14件大事和改善民生的28件实事，攻坚克难，锐意进取，圆满完成了市二届人大四次会议确定的各项目标任务，实现了“十二五”的良好开局。预计全年完成地区生产总值126亿元，同比增长14.2%;完成地方财政一般预算收入7.8亿元，增长48.6%;完成全社会固定资产投资130亿元，增长36%;城镇居民人均可支配收入达到14500元，增长11.2%;农民人均纯收入达到4100元，增长18%。城镇登记失业率控制在4.18%以内，人口自然增长率控制在11.36‰以内，万元GDP能耗控制在1.687吨标煤以内。地区生产总值、财政一般预算收入、固定资产投资、规模以上工业增加值、农民人均纯收入五项指标增幅名列全区前茅。

　　一、坚持以结构调整为主线，产业转型进程加快

　　工业经济实现突破。加快煤炭、岩盐、风能等资源转化利用，提升工业化发展水平，预计完成工业增加值18亿元，增长36%。园区建设步伐加快，盐化工循环经济扶贫示范区10平方公里征地拆迁及主次干道等基础设施建设基本完成，累计完成投资3.05亿元;原州区通用航空产业园、西吉闽宁产业园、隆德六盘山产业园、泾源轻工业产业园基础设施建设全面推进。重点工业项目进展顺利，实施工业项目69项，完成投资26.2亿元，年产700万方采输卤、年产100万吨真空制盐及氯化烷烃等项目开工建设;王洼二矿年产150万吨原煤项目建成投产，银洞沟年产300万吨原煤项目完成主体工程;华电西吉月亮山风电一期49.5兆瓦项目并网发电，宁夏发电集团10兆瓦光伏发电项目开工建设;三鼎公司年产2万吨马铃薯精淀粉生产线建成投产，雨润集团彭阳帝元10万头肉牛屠宰加工项目开工建设。工业效益逐步显现，全年完成发电量40亿度、生产原煤300万吨，累计生产原油36.9万吨，生产马铃薯精淀粉11万吨、牛羊肉系列产品7500吨、中药饮片1500吨、水泥制品89万吨。招商引资成效显著，实施引进项目150个，实际到位资金52.11亿元，增长77.8%。节能减排全面完成了自治区下达的任务，二氧化硫和化学需氧量排放量分别控制在1.04万吨和2.2万吨以内。

　　农业经济稳步发展。认真落实强农惠农政策，深入实施农民收入倍增计划，力促草畜、马铃薯、设施农业等特色优势产业提质扩量，预计完成农业增加值33亿元，增长6%。狠抓粮食生产，粮食总产量稳定在70万吨以上。草畜产业以实施百村肉牛养殖示范工程为抓手，更新改良多年生牧草30万亩，新建肉牛养殖示范村30个、存栏200头以上肉牛养殖园(区)10个，肉牛饲养量达到82.26万头，增长5.5%。泾源县被自治区确定为宁夏优质肉牛养殖示范县，泾河系列牛肉产品取得有机认证。马铃薯产业以三级脱毒种薯繁育推广体系建设为重点，生产原原种1.04亿粒，种植种薯22.16万亩，建立万亩马铃薯抗旱增产基地11个，示范田亩产首次突破万斤大关，全市播种马铃薯210.6万亩，总产量210万吨。设施农业以创建自治区级和市级现代农业示范基地为带动，新建设施农业5.8万亩，建设设施农业示范区50个，完成以秋覆膜为主的旱作节水农业105.5万亩，设施农产品总产量达到56.7万吨，冬菜北上、夏菜南下的流通格局基本形成。枸杞、西芹、辣椒、苗木、中药材、菌草、花卉等区域特色产业快速发展，原州区被命名为“中国(西部)冷凉蔬菜之乡”，西吉县着力打造葫芦河川道区百公里特色蔬菜产业带，隆德县着力打造六盘山道地中药材和花卉产业，泾源县苗木种植规模扩大，“彭阳辣椒”获得国家地理标志认证。大力推进农业产业化经营，新流转土地13.7万亩，引进雨润、佳立等大企业、大公司参与优势特色产业开发建设。积极兴办各类专业合作经济组织，拓宽农产品市场，健全农产品质量安全体系。加快实施品牌战略，六盘山农产品品牌获得自治区著名商标。

　　第三产业提速发展。大力发展旅游、物流、商贸等服务业，积极培育新的经济增长点，预计完成第三产业增加值58亿元，增长8%。加快旅游资源开发，须弥山博物馆建成开放，完成须弥山、六盘山国家森林公园旅游道路等基础设施建设，火石寨、老龙潭旅游基础设施建设进展顺利。组建六盘山旅游集团公司，初步理顺了旅游产业开发管理体制。成功举办了第七届六盘山山花旅游节和“六盘山旅游”西安、兰州、银川推介会，宁夏公共卫视每天播放六盘山旅游宣传片，六盘山旅游知名度进一步扩大。全年接待游客170万人次，实现旅游社会总收入6.3亿元，分别增长12%和8%。积极培育现代市场体系，编制完成《固原市现代物流产业规划(2010—2020)》，启动建设西兰银综合物流园区。加快实施“万村千乡”市场工程、社区蔬菜直销网络体系建设，建成万国商业文化广场、瑞丰一级农产品综合批发市场等一批商贸设施和30个社区蔬菜直销店。房地产、住宿餐饮、交通运输、家政服务、信息咨询等服务业快速发展。银行保险等金融业健康发展，全市各项存款余额193亿元，贷款余额110亿元，金融支持地方经济社会发展力度增大。全面落实国家鼓励扩大消费政策，认真实施家电汽车摩托车下乡和家电以旧换新工程，预计全市实现社会消费品零售总额37亿元，增长15%。大力实施“菜篮子”工程，加强价格监管，建立了物价调控基金，保障了市场供给和物价基本稳定。

　　二、坚持以生态移民为重点，扶贫攻坚卓有成效

　　生态移民实现良好开端。把生态移民作为全市的“一号工程”，以“搬得出、稳得住、逐步能致富”为目标，举全市之力强力推进。县内移民着力在科学规划、产业支撑、政策落实、加快进度、确保质量上下功夫，采取“七个一”建设模式，投资6.5亿元建成水、电、路、通讯、学校、卫生、商贸等公共服务设施配套的安置区(点)43个，建成生态移民安置房4436套、劳务移民周转房1184套，完成移民4252户17948人;加大移民产业扶持力度，配套建设了养殖圈棚、日光温室、大中拱棚等生产设施，开展技能培训，促进移民稳定增收。县外移民着力在主动对接、加强协调、督促落实上下功夫，建立了与迁入地的联席会议制度，确定了搬迁对象，确认了移民安置方式，组织群众搬迁定居，县外22个安置点建成生态移民安置房6966套、劳务移民周转房3639套，完成移民1352户4650人。

　　扶贫攻坚取得明显成效。编制了《六盘山区集中连片特殊困难地区扶贫攻坚规划》，通过多方努力争取六盘山区被国家确定为今后十年扶贫攻坚主战场。加大整村推进、产业扶贫和互助资金投放力度，实施“扶贫到户、责任到人”攻坚工程，扎实做好闽宁对口扶贫协作和社会扶贫工作。共整合资金3.86亿元，完成132个整村推进扶贫村开发年度任务，新增互助村84个，极大改善了421个贫困村的生产生活条件，全年贫困人口减少5.4万人。

　　三、坚持以建设区域中心城市为目标，城乡面貌显著变化

　　宁南区域中心城市建设步伐加快。修编完成了《固原市城市总体规划(2011—2030)》，拉开宁南区域中心城市暨大县城建设大会战序幕。扎实开展“城市基础设施建设年”活动，集中实施城市重点建设项目30项，完成投资6.7亿元。新建改建六盘山路、南城路、北关路、中山北街等市区道路13条16.4公里，改造硬化14条背街巷道，配套建设供水管网12.4公里、集污管网15公里、供热管网53.4公里，新建改造换热站9座，完成了热电联产市区供热并网工程。新区开发建设步伐加快，市应急指挥中心、公安局等行政设施投入使用，市检察院、国土局、住建局迁建工程开工建设，派胜世茂城、华福御景天城、盛世华都、华祺酒店、新天地商业广场等一批商住商贸项目和试验区服装城、家具城、汽车城、农机市场等一批重点项目进展顺利。启动争创自治区园林城市活动，新增绿地644.96亩。加大城市管理综合执法力度，依法拆除违法建筑6.72万平方米，集中开展整治“脏乱差”专项行动，市容市貌明显改善。

　　大县城、中心镇、新农村建设协调推进。把大县城建设与地域风情、民族传统和时代精神结合起来，四县县城在扩容提质上形成了一批新亮点、新景观，西吉县城道路和文化设施建设水平明显提升，隆德县城道路改造、古柳公园二期等旧城改造项目顺利实施，泾源县“一城三区两园”城市框架全面拉开，彭阳县城悦龙山广场、安定大桥等建设项目基本完成。积极打造特色小城镇，以原州区三营镇为代表的10个中心镇建设稳步推进。扎实开展新农村建设，建成社区化新农村示范村20个。

　　四、坚持以重大项目为支撑，发展后劲明显增强

　　道路电力通信建设成效显著。福银高速市境段全线贯通，兰青高速东山坡至毛家沟段建设进展顺利，原州区至王洼运煤铁路专线、309国道市境段改造全线开工，101省道市区段改造前期工作顺利进行，新修农村水泥沥青公路1014公里。大力推进城市配电网建设和农村电网改造升级工程，建成王洼110千伏输变电工程、西吉月亮山风电一期110千伏输变电工程，电力保障能力进一步增强。扩大移动通信基站建设，实现了3G网络全覆盖。实施光纤到楼工程，网络承载能力增强，信息化服务支撑能力明显提升。

　　农田水利建设不断加强。实施百万亩库井灌区高效节水农业示范工程，新增节水灌溉面积2.14万亩，改善灌溉面积2.24万亩。新修旱作基本农田12.8万亩。固原地区城乡饮水安全水源工程全面启动，秦家沟水库具备蓄水条件，秦东补水工程完成前期征地工作，中庄水库、龙潭水库建设项目完成招投标工作;彭堡水源地地下截潜水库完成地下连续墙2000米;开工建设病险水库除险加固工程15座、农村饮水安全工程13处。

　　生态环境建设纵深推进。以大六盘水源涵养林建设为重点，完成人工造林21万亩、封山育林14.8万亩、城乡大环境绿化3.13万亩、道路绿化439公里。完成退耕还林补植补造87.4万亩，2003年度完成的60万亩退耕还林顺利通过国家验收。严格责任追究，封山禁牧工作进一步加强。完成长城梁绿化一期工程1.2万亩，建成须弥山“一河两山”等3000亩以上集中连片绿化点6处。治理小流域17条，兴建小型水保工程266座，治理水土流失面积382平方公里。全力实施水污染防治和农村环境综合整治，建成生态示范乡(镇)5个、生态示范村50个。

　　五、坚持以改善民生为根本，和谐社会建设稳步推进

　　劳务及促进就业工作深入开展。培育提升“六大劳务”品牌，新增劳务基地50个，成功举办第二届农民工“大练兵、大比武” 技能竞赛活动，完成农村劳动力转移就业培训1.39万人次，实现农村劳动力转移就业30.55万人，劳务总收入23.22亿元，农民人均劳务收入1850元，增长20%。认真实施各项就业工程，大学生就业3036人，开发公益性岗位500个，新增城镇就业8333人，实现了零就业家庭动态清零。扎实开展“六大创业行动”，提升创业孵化示范园区7个，发放小额担保贷款1.75亿元，扶持创办小企业445个，培养小老板658人，创造新岗位4029个。

　　社会保障体系不断完善。全面启动新型农村养老保险制度，参保率达到87.5%。在全区率先建立了市级统筹城乡医疗保险制度，全市范围实现了“一卡结算”服务，参保率达到95%以上。城镇职工养老、失业、工伤、生育保险覆盖面进一步扩大。社会保障“一卡通”工程全面启动。为1868名市区失地农民办理城市养老保险。不断完善社会救助机制，城乡低保标准进一步提高，全市纳入城乡低保达22万人，为1.1万名80岁以上的城乡低收入家庭老人发放了高龄津贴，市本级为全市城乡低保户每户发放70元和50元生活补贴。住房公积金归集总额达到15.88亿元，贷款总额达到10.48亿元。大力推进保障性安居工程，新建廉租房、经济适用房等保障性住房68.47万平方米，改造农村危窑危房1万户，1.3万户城乡低收入家庭喜迁新居。实施宋家巷民族特色商住区二期工程，受益群众1700户6800人;将城中村改造与生态移民有机结合，新建居民安置点3个。社会福利和社会慈善事业健康发展，市老年活动中心建成使用。

　　社会各项事业不断进步。加快发展教育事业，实现教育强市创建目标，基本普及高中阶段教育工作深入实施。投资2.7亿元的固原一中迁建工程交付使用。开工新建中小学7所、乡级中心幼儿园11所，完成中小学校舍安全工程33.9万平方米。教育教学质量进一步提高，中考总分及格率达到68%，高考录取率达到71%。认真落实教育普惠政策，全面实施中小学生营养早餐工程，开展营养午餐试点工作，积极资助贫困高中生、中职生、大学生完成学业。加快职业教育发展，固原民族职业技术学院、西吉职中、隆德职中等扩建工程进展顺利。大力推进科技创新，加大科技投入力度，实施科技推广项目91项;科技特派员达到523名，实现了特色优势产业科技服务全覆盖，原州区被评为全国科普示范县(区)。积极实施人才发展规划纲要，人才队伍建设得到加强。深入实施文化惠民工程，建成“农家书屋”515家。积极开展精神文明创建活动，涌现出白志林等一批先进典型，评选出十大道德模范。开展建党90周年“红歌唱响六盘山”系列活动。整理完成了《固原市非物质文化遗产名录》。西吉县被命名为中国首个“文学之乡”，原州区被授予“全国民间文化艺术之乡”。积极开展文化下乡，编排具有地方特色的文化剧目，举办了一系列丰富多彩的文体活动。完成广电中心主体工程建设、1万户广播电视“村村通”建设任务和全国直播卫星公共服务“户户通”试点工作。不断提升公共卫生服务水平，市人民医院通过三级医院评审验收，原州区、隆德县医院通过二级甲等医院评审验收，县级公立医院改革试点工作进展顺利，地方病防治工作得到加强。市中医院迁址扩建全面完成，为全市所有村卫生室配备了新十件医疗设备，基层医疗卫生基础设施条件显著改善。深入开展人人享有基本医疗卫生服务，实现了全市城市社区、乡镇卫生院、村卫生室药品零差率销售。狠抓计生工作不放松，实施少生快富工程3403例，“硝沟模式”在全区推广，全市人口出生率控制在16.6‰以内，同比下降1.08个千分点;出生政策符合率达到 84.6%，上升1.4个百分点。圆满完成第六次全国人口普查工作。外事侨务工作得到加强，与俄罗斯加里宁格勒市建立了友好城市关系。妇女儿童“两纲”终期评估通过国家验收。地震、人防、文物、档案、地方志、统计、供销、气象、残疾人等工作取得新的成绩。

　　社会管理创新工作得到加强。制定了《关于加强和创新社会管理的实施意见》，建立健全管理服务机制。大力推进平安固原建设，深入开展矛盾纠纷和不稳定因素排查化解，严厉打击各类刑事犯罪，切实提高群众的安全感。不断完善大接访制度，妥善解决群众合法合理诉求，化解信访积案21件，化解率85.7%。集中开展了道路交通、矿山、危险化学品、人员密集场所、建筑施工和食品安全等重点领域和关键行业的排查整治，安全生产形势总体平稳。城乡社区建设有序推进，新创建星级社区6个。进一步加强军政军民团结，深入推进双拥共建。切实加强民族团结，依法管理宗教事务，坚决抵御和严厉打击境内外非法宗教势力渗透破坏活动，确保了社会和谐稳定。

　　六、坚持以效能建设为抓手，政府自身建设不断加强

　　深入开展“思想大解放、树立新形象”活动，广大干部抢抓机遇、发挥优势、破解难题的意识和能力进一步增强。围绕“转型发展开好局，创新驱动起好步”这一要求，提高执行力，奋力抓落实，政府效能进一步提升。健全了重大事项专家咨询、社会听证和新闻发布制度，聘请知名专家参与编制重大规划、论证重点项目、开展专题辅导和决策咨询;邀请人大代表、政协委员列席政府常务会议，科学化、民主化、法制化决策机制进一步完善。多层次推进政务公开，在固原政府网站开通了“政民互动”栏目，网络办理群众咨询问答近1000件。深入推进依法治理，启动“六五”普法宣传教育，自觉接受人大依法监督、政协民主监督、群众评议监督和社会舆论监督，办理人大代表建议23件、政协提案166件，办结率100%。完成水务一体化改革，集体林权制度和旅游运营体制改革有序推进，泾源县、彭阳县被评为“全国林改典型县”。严格落实“十项规定”、“八办要求”，加大行政问责力度，干部作风进一步转变，发展环境进一步优化。认真落实党风廉政建设责任制，制定了《政府投资项目建设管理办法》，加强重点行业、重点领域、重大项目资金管理使用的审计监督和行政监察，廉政建设取得了新成效。

　　各位代表，过去的一年，我们坚定信心、奋力拼搏，战胜了前进道路上的各种困难，取得了令人鼓舞的成绩，全市经济社会发展已步入了一个新的阶段。这些成绩的取得，是自治区党委、政府和市委正确领导的结果，是市人大、市政协监督支持的结果，是全市广大干部群众共同努力的结果。在此，我代表市人民政府，向支持政府工作的各位代表和委员，向辛勤工作在全市各条战线上的全体劳动者，向各民主党派、工商联、人民团体和社会各界人士，向中央、自治区驻固各单位、人民解放军、武警消防官兵和公安干警，向所有关心固原建设和发展的朋友们，表示崇高的敬意和衷心的感谢!

　　在肯定成绩的同时，我们也清醒地认识到，全市经济社会发展中仍然存在一些突出问题，主要表现在：经济总量小，结构不合理，产业层次低，资源转化慢;工业经济薄弱，缺少骨干企业和大工业项目，招商引资和融资困难;扶贫开发任务仍然艰巨，农民持续增收难;城市化水平低，城市功能不完善，服务业发展活力不足;社会事业滞后，就业和人口压力大，社会保障体系尚需完善;政府职能还需进一步转变，发展环境还需进一步优化，干部作风还需进一步改进。对此，我们将在今后的工作中高度重视，采取有力措施，切实加以解决。{CACOPAGE}

　　2012年主要目标任务

　　2012年是实施“十二五”规划承上启下的关键之年，是国家启动实施六盘山集中连片特困地区扶贫攻坚的第一年，是经济社会发展面临机遇与挑战并存的一年，也是固原撤地设市10周年，做好明年的工作，具有十分重要的意义。我们将紧紧围绕市第三次党代会确定的“跨越发展、强市富民”主题和“团结、鼓劲、创新”的要求，勇于担当，埋头苦干，稳中求快，乘势而上，全面完成经济社会发展的各项目标任务，向全市人民交上一份满意的答卷。

　　2012年政府工作的总体要求是：深入贯彻落实科学发展观，认真贯彻落实自治区党委十届十四次全体(扩大)会议、全区经济工作会议和市第三次党代会精神，以跨越发展、强市富民为主题，以稳中求快为总基调，以实施“十二五”规划和西部大开发战略为总揽，按照团结、鼓劲、创新的要求，着力推进经济发展方式、社会管理方式、思想观念和工作作风“三个转变”，在实施生态移民攻坚、工业强市、特色农业增效、中心城市带动、文化旅游品牌、重大项目带动“六大战略”上取得新突破，全面加强经济、政治、文化、社会建设，争创西部地区生态文明示范区、民族团结示范区、扶贫开发示范区，争取使各项工作在全国有亮点，西部有位置、宁夏勇争先，努力建设新固原，以优异成绩迎接自治区第十一次党代会和党的十八大胜利召开。

　　2012年全市经济社会发展主要预期目标为：全市地区生产总值达到144亿元，增长14%以上;地方财政一般预算收入力争突破10亿元大关，增长25%;全社会固定资产投资完成166亿元，增长25%以上;城镇居民人均可支配收入达到16240元，增长12%;农民人均纯收入达到4610元，增长12.5%。城镇登记失业率控制在4.4%以内;人口自然增长率控制在11‰以内;万元GDP能耗控制在1.687吨标煤以内。

　　为完成上述目标任务，2012年着力抓好事关经济社会发展的15件大事和改善民生的30件实事，主要做好以下九个方面的工作。

　　一、突出资源开发转化，实现工业经济新跨越

　　坚定不移地实施工业强市战略，紧紧抓住国家扶贫攻坚重大产业项目优先向连片特殊困难地区布局的历史机遇，着力培育壮大煤电油、盐化工、特色农产品加工业和战略性新型产业四大产业，努力做大工业总量，加快新型工业体系建设。实现工业增加值22亿元，增长22%。

　　强力推进产业园区建设。按照产品项目、公用辅助、物流传输、环境保护、管理服务“五个一体化”的开发模式，高起点规划、高标准建设盐化工循环经济扶贫示范区，基本完成“七通一平”等基础设施建设，建成布局合理、配套完善、功能齐全的自治区级循环经济扶贫示范区。优化固原扶贫发展试验区布局，抓好原州区长城产业园、西吉闽宁产业园、隆德六盘山产业园、泾源轻工业园的科学规划，搞好产业定位，优化产业布局，形成产业聚集，不断增强吸引力和竞争力。

　　强力推进重点项目建设。加快年产700万方采输卤、年产100万吨真空制盐及氯化烷烃系列产品等项目建设进度。启动王洼千万吨级矿区建设，建成银洞沟年产300万吨原煤生产项目，开工建设王洼煤矿年产600万吨技改扩建、600万吨洗选煤厂项目。加快发展风力发电、光伏发电等新能源产业，西吉月亮山风电二期49.5兆瓦风电项目并网发电，积极推进三、四期49.5兆瓦风电项目建设;完成宁夏发电集团10兆瓦光伏发电项目建设，全力配合做好六盘山热电厂2×670兆瓦和国电英力特2×350兆瓦自备电厂前期各项工作。支持彭阳石油资源勘探开发和转化增值。力争开工建设日产4500吨水泥熟料生产线及配套9.0兆瓦纯低温余热发电技改项目。千方百计，创造一切条件，争取中国铝业集团公司100万吨煤电铝项目落户固原。

　　强力推进传统产业升级。马铃薯淀粉加工业要通过淘汰落后产能，关闭污染企业等方式，引导企业联合重组，促进产业集聚，全面提升加工能力和水平。草畜加工业要加快雨润集团彭阳帝元10万头肉牛屠宰加工项目建设进度，力促泾河食品、单家集牛羊肉、隆昊肉类及荣发草业公司达产达效。中药材加工业要加快明德公司年产5000吨中药饮片等项目建设步伐，积极开发药品系列产品，不断提高市场竞争力。粮油加工等行业也要加快技术改造步伐，扩大产能，提高效益。

　　强力推进招商引资工作。抓住自治区建设内陆开放型经济区的机遇，依托中阿经贸论坛、闽宁对口协作、陕甘宁革命老区联席会议等平台，加强与银川、宁东基地和周边省市区域合作和企业合作，主动对接东部产业转移，完善招商机制，创新招商方式，拓宽招商领域，年引进实际到位资金40亿元以上。引进一批产业链条长、带动力强的战略投资项目，着力在引进中央骨干企业和国内或行业500强企业上取得突破。

　　二、突出特色农业增效，力促农业经济新突破

　　认真落实各项强农惠农富农政策，加快农业科技进步，保持粮食生产基本稳定，大力发展优势特色产业，努力促进农业增产、农民增收、农村发展。实现农业增加值35亿元，增长6%。

　　着力推动优势特色产业提质增效。围绕建设多元化饲草基地、良种基础母畜扩繁、科学化规模化养殖三个重点，新培育肉牛养殖示范村20个，新建存栏200头以上养殖场(园)15个，肉牛饲养量达到86万头。狠抓优化品种结构、种薯质量监管、一级种薯推广种植三个环节，建设60个种植业示范园区(基地)，推进马铃薯三级脱毒种薯繁育推广体系建设，繁育原原种1.1亿粒，建设种薯繁育基地24.4万亩。按照提高质量、提升效益、打造品牌的三个要求，新建设施农业2.6万亩，维修改造日光温室1.4万亩，完成以秋覆膜为主的旱作节水农业100万亩。支持中药材、小杂粮、油料、苗木、林果、枸杞、花卉、菊芋等区域特色产业发展。

　　着力增强农业综合生产能力。实行严格的耕地保护制度，积极争取国家农业综合开发和土地整理项目，切实提高耕地产出能力。以23个自治区级和27个市级现代农业示范基地为平台，建设一批集繁育制种、科技示范、科普教育、信息交流为一体的现代农业示范园。深化科技特派员创业行动，充分发挥国家马铃薯改良中心等科研机构的科技优势，引进、研发和推广一批新品种、新技术、新装备。加强人工影响天气工作，增强抵御自然灾害能力。

　　着力提升农业产业化水平。完善农业产业化龙头企业扶持激励机制，每个县(区)新引进或发展1家投资5000万元以上的农业产业化龙头企业，积极引导土地有序流转，适度发展规模经营。支持发展科技、信息、农资、农产品购销服务等各类专业合作经济组织，每个县(区)至少发展4家。实施品牌战略，进一步培育六盘山农产品品牌，提高市场竞争力。健全农产品质量安全体系，做好重大动物疫病防控工作。

　　三、突出旅游物流业发展，再造第三产业新优势

　　依托区位优势，加快建立现代服务业体系，推动服务业总量扩张，结构优化，产业升级。实现三产增加值64亿元，增长10%。

　　大力打造特色旅游业。修编完成《固原市旅游发展总体规划》。支持六盘山旅游集团公司整合资源，规范运行，提升效益。全面完成老龙潭、火石寨景区旅游基础设施建设，开工建设须弥山至火石寨旅游通道，重新对六盘山红军长征纪念馆布展，完善六盘山红色景区功能，提高吸引力。拓展和挖掘凉殿峡、大雪山、白云寺、安西王府等文化旅游资源。加大宣传促销力度，提升六盘山旅游知名度。加快推进旅游产品的多元化，推出一批个性化的产品。将旅游与生态、爱国主义教育、文化、民俗有机融合，精心打造精品景区和旅游线路。打响“高原绿岛”、“长征圣山”、“丝路重镇”、等品牌，构建独具特色的旅游目的地。全年接待游客200万人次以上，实现旅游社会总收入7亿元以上。

　　大力发展现代物流业。加快西兰银物流园区建设，投资15.29亿元完成园区征地拆迁和“七通一平”基础设施，按照园区规划布局，通过市场化运作，引进大企业分区分类建设，开工建设物流建材城项目。加快清水河综合物流园、火车站农产品物流园、盐化工示范区工业物流园和各县城综合物流中心规划及建设。

　　大力提升现代服务业。实施好“万村千乡”市场工程，改造提升农家店150个，配送中心3个，农贸市场4个。加快社区蔬菜直销网络体系建设，新建直销店20个。培育和繁荣城乡市场，扩大城乡居民消费。实现社会消费品零售总额42.5亿元，增长15%。继续推进“菜篮子”工程建设，确保市场供给。加强金融生态环境建设，调整信贷结构，大力支持“三农”和中小企业发展。培育壮大物业管理、中介服务、信息咨询、家政服务、商务会展、保险担保等新兴服务业。改造提升交通运输、餐饮住宿等传统服务业。

　　四、突出生态移民攻坚，谱写扶贫开发新篇章

　　坚持把生态移民作为从根本上解决贫困问题、同步实现全面小康的重大举措，举全市之力继续打好攻坚战。

　　全力推进生态移民攻坚工程。以群众满意为最高标准，全面完成14698户62935人生态移民任务，确保“搬得出、稳得住、逐步能致富”。县内移民要坚持规划引领、基础先行、产业支撑、教育引导、服务跟进、廉洁移民的原则，将住房与产业发展、基础设施配套、社区化服务管理教育相结合，完成移民4614户19439人。县外移民要积极主动对接，跟踪做好协调、组织、管理、服务、教育等工作，完成移民10084户43496人。

　　全力打好扶贫开发攻坚战。继续坚持开发式扶贫方针，完善并认真实施六盘山集中连片特困地区扶贫攻坚规划。认真实施“双到”扶贫攻坚工程。全面完成132个整村推进扶贫年度任务，加大产业扶贫、移民扶贫、科技扶贫、项目扶贫力度。扩大互助资金扶持面，新增80个互助资金项目村。大力实施“雨露计划”培训工程，增强贫困人口自我发展能力。进一步加强社会帮扶力度，拓宽定点帮扶领域，提升闽宁对口帮扶协作水平，促进扶贫开发向工农并重多产业协调发展转变。

　　五、突出中心城市带动，建立城乡发展新格局

　　狠抓扩大规模、完善功能、提高品位、产业支撑四个关键，推进区域中心城市和大县城建设，形成以市区为核心、四县县城为骨干、中心镇为支撑的宁南城镇集群，不断提高城市吸引力和辐射带动能力，促进城乡统筹发展。

　　扎实抓好区域中心城市建设。突出“生态文化旅游”城市定位，按照“改造老城区、完善新市区、开发西南区”的思路，以更大的气魄、超常的举措，实现新突破，展示新形象。坚持以规划为龙头，按照新修编的《固原城市总体规划》，完成道路、供排水等专项规划、控制性详规和部分修建性详规，维护规划的严肃性和权威性。规划建设一批纪念撤地设市10周年标志性工程。开工建设省道101线市区过境段改线和与中山北街连接线等9条道路36.25公里，改建政府街等街道3.4公里，配套新建改建供排水管网13条22.23公里、供热管网33.52公里，建设改造换热站10座，建设垃圾渗滤液处理工程。积极争取固原市污水处理厂二期、第三水厂和博物馆广场等项目立项建设。继续加快尚都国际、派胜世茂城、华祺酒店等一批商贸商住、星级酒店建设，启动上陵商业广场建设项目，完成东海南街等5个棚户区改造。推进数字固原地理信息框架建设，提升城市信息化管理水平。

　　快速推进大县城、中心镇和新农村建设。突出西吉民族特色、隆德文化特色、泾源旅游特色、彭阳生态特色，实施一批基础设施建设项目和产业开发项目，使之成为承接区域中心城市产业辐射、配套和带动小城镇发展的重要引擎。加快中心镇规划和基础设施建设，每县(区)建设1-2个特色鲜明的中心镇。加快新农村规划和建设，突出产业支撑和基础设施配套，建设社区化新农村示范村20个。以城乡结合部和景区周边农村为重点，加快城镇基础设施向农村延伸。

　　切实加大城市管理和城乡环境整治力度。深入开展“环境卫生综合整治和违法建筑查处年”活动，创新城市管理机制，开展以环境卫生、违法建筑、交通秩序、规范市场为重点的专项整治活动，加大综合执法力度，健全长效机制，提升城市管理水平。加强园林绿地建设与管理，积极开展园林式小区、园林式单位的创建活动，实现自治区园林城市创建目标。继续开展城乡环境综合整治活动，加快公用设施建设改造步伐，实施净化、绿化、美化、亮化工程。

　　六、突出生态建设与保护，构筑西部生态新屏障

　　坚持经济发展与生态环境建设并重，因地制宜，整体推进，加快构筑西部生态安全屏障。

　　狠抓生态建设。争取启动国家西部地区生态文明示范城市试点工程。全力实施“三河源”保护、三北防护林二期和大六盘生态经济圈建设工程。争取启动清水河湿地公园建设，规划好中庄水库水源地生态保护项目;加大禁牧封育力度，完成退耕还林补植补造30万亩、营造林30万亩;完成城乡环境绿化2万亩、道路绿化200公里。综合治理水土流失面积200平方公里，各县(区)至少建设1个3000亩以上集中连片生态示范点、3至5个移民新村绿化示范点。

　　狠抓环境保护。以污染物总量控制为主要手段，加强城乡水源地保护和水污染防治，加强农村环境综合整治和生态环境保护工作，加强环境监管，切实解决群众关心的环境突出问题。加大马铃薯淀粉废水治理技术研究和综合利用技术推广运用。实施农村环境连片整治项目18个、农村集中式饮用水源地保护项目25个。主要污染物排放量控制在自治区下达的年度指标以内。继续抓好公共节能，树立全社会节能环保意识。

{CACOPAGE}

　　七、突出重点项目建设，增添跨越发展新动力

　　始终把重点项目建设作为跨越发展的第一推动力，全力争取、引进和实施一批大项目、好项目，以项目建设推动经济社会跨越发展。

　　抓好农田水利项目建设。新增旱作基本农田12万亩。加快固原地区城乡饮水安全水源工程建设步伐，新建中庄水库，加固龙潭水库，续建秦家沟水库至东山坡引水连通工程。建设彭堡水源地地下截潜水库。完成14座病险水库除险加固，新建12处农村饮水工程，解决10万农村人口饮水安全问题。加快实施百万亩库井灌区节水改造项目，新增节水灌溉面积2.4万亩，新增灌溉面积1.3万亩，改善灌溉面积2.4万亩。采取综合措施治理臭水河苦咸水排放。

　　抓好道路交通项目建设。强化协调服务，完成原州区至王洼运煤铁路专线、兰青高速东山坡至毛家沟段、309国道固原市境段改造的年度建设任务，开工建设西吉至静宁一级公路。加大县道、乡道、村道建设力度，新建农村沥青(水泥)公路1000公里、农村客运招呼站70个。改革市区公共交通管理体制，优化资源配置，逐步建立政府主导的市区公共交通体系，提升公共服务的能力和质量。

　　抓好电力通信项目建设。进一步完善110千伏电网网架结构，新建申庄110千伏变电站和110千伏高平输变电工程，满足盐化工循环经济扶贫示范区和市区发展用电需求。实施35千伏及以下电网建设项目，改造升级农村电网。抓好移动网络建设和优化工作，进一步扩大3G网络覆盖面。实施全市无线数字城市建设项目，积极推进“三网融合”进程。实施宽带提速工程，加快行政办公、企业商务、城乡社区、居民生活等各个领域信息化服务平台建设，提升全市信息化水平。

　　八、突出基本公共服务，推动社会事业新发展

　　坚持统筹兼顾，加快教育科技、文化卫生等社会事业发展，促进经济增长与社会发展相协调。

　　继续坚持教育优先发展。加强教育基础设施建设，全面完成中小学、幼儿园在建任务，扩建固原二中、原州五中、西吉回中、西吉三中、隆德一中、彭阳一中和泾源高级中学等7所高中，完成中小学校舍安全工程20万平方米。实施公共义务教育阶段校舍标准化建设和薄弱学校改造项目，促进义务教育均衡发展。西吉县、泾源县基本普及高中阶段教育工作通过自治区评估验收，全市实现基本普及高中阶段教育目标。加快发展学前教育，坚持公办民办并举，扩大学前教育资源。加快发展职业教育，培养适应市场和我市产业发展的实用型、技能型人才。加快发展特殊教育、民族教育。实施基础教育质量提升工程，统筹培训城乡教师。加快教育信息化建设步伐，促使教育发展由规模数量扩张向质量效益提升转变。加强科普教育，提高全民科技素质。深入开展科技特派员创业行动和科技服务生态移民工作，加快促进科技成果转化运用，大力推广新品种、新技术、新设备。努力实施“六盘英才”等重点人才项目，统筹推进人才队伍建设。

　　继续推进文化固原建设。认真贯彻落实《中共中央关于深化文化体制改革、推动社会主义文化大发展大繁荣若干重大问题的决定》，大力发展繁荣文化事业。全面完成固原市图书馆建设，加快固原市广电中心建设，争取立项建设固原市文化馆，完善直播卫星公共服务户户通工程。将文化建设与文学、文艺、旅游、体育事业发展相结合，推出一批新的西海固文学作品，筹办第二届六盘山登山节、须弥山佛教文化艺术节和“百乡千村农民体育月”等文体活动，提升《王洛宾的花儿情》剧目，继续做好送戏下乡、农村数字电影放映等工作，扩大公共文化服务覆盖率。加快文化产业发展，积极扶持非公中小文化企业，鼓励社会资本进入文化创意产业领域，培育一批文化企业，鼓励支持利用现有文化体育设施开展有偿文体服务活动。

　　继续完善公共卫生医药服务体系。加快推进医药卫生体制改革，探索建立符合固原实际的医药卫生服务体制，促进基本公共卫生服务均等化。开工建设县(区)卫生监督所，新建标准化村卫生室100所，积极争取市人民医院内科大楼立项。继续做好重大传染病防控、突发公共卫生事件应急处置工作，深入实施人人享有基本医疗卫生服务和“万名医师支援农村卫生工程”，不断提高城乡群众健康水平。

　　继续加强人口与计划生育工作。狠抓人口和计划生育工作不放松，完善利益导向机制和奖励扶助政策。提高优质服务能力，提高人口素质。严格目标责任管理，大力推广“硝沟模式”，全面实施“少生快富”工程整村推进，积极开展“星级乡镇”和“少生快富”示范户创建活动。全市人口出生率控制在16.77‰以内，出生政策符合率达到86.5%以上。

　　九、突出民生改善和保障，开创和谐社会新局面

　　坚持把经济增长与改善和保障民生相结合，创新社会管理，健全服务体系，解决好群众最关心、最直接、最现实的利益问题。

　　进一步实施劳务及促进就业工程。加快推进农村劳动力转移就业，继续开展农民工技能培训和“大练兵、大比武”活动，完成职业技能培训8000人、职业技能鉴定6400人;进一步扩大有组织、技能型劳务输出，稳定转移农村劳动力30万人，劳务总收入达到25亿元以上。实施更加积极的就业政策，多渠道开发就业岗位，围绕解决高校毕业生、城镇困难群众等就业，加大就业扶助和服务力度，新增城镇就业6500人以上。大力推进全民创业行动，加快创业园区建设，新培育小企业300个，培养小老板300个，发放创业小额担保贷款1.5亿元以上，带动创业和就业3000人，争取创建1个全国西部地区农民创业促进工程试点县。

　　进一步完善社会保障体系。不断扩大养老等各类社会保障范围，提高统筹层次和保障水平，落实好各项保障措施和救助机制。加快推进统筹城乡居民医疗保险和统筹城乡居民养老保险，参保率分别达到93%和80%以上。认真落实市区失地农民最低生活保障制度。全面推进社会保障“一卡通”建设，力争全市发放社会保障卡80万张。切实完善社会救助体系，加大对低收入群众和五保老人、重度残疾人、孤儿等特殊群体的补贴和救助力度。进一步扩大住房公积金制度覆盖面。多方筹集配套资金，完成廉租房、公共租赁房等保障性住房40.76万平方米，实施农村危窑危房改造8000户，着力解决城乡低收入家庭住房困难问题。

　　进一步加强和创新社会管理。深入推进平安固原创建活动，提升社会管理服务水平，切实维护社会和谐稳定。建立健全社会稳定风险评估机制、群众诉求表达机制、矛盾预防化解机制，统筹协调各方面利益关系，切实维护群众合法权益。高度重视信访工作，深入开展矛盾纠纷“大排查、大接访、大调处、大防控”活动，妥善解决群众合法合理诉求，最大限度把矛盾解决在基层、化解在萌芽状态。加强城乡社区建设，增强社区服务功能。强化流动人口和特殊人群、非公有制经济组织和社会组织的管理服务。加强公共安全体系建设，着力提高防控和应急处置能力。扎实开展“六五”普法，提升依法治市水平，促进公平正义。加强社会治安综合治理，强化社会治安防控体系建设，严厉打击各类刑事犯罪，切实提高群众的安全感。严格落实安全生产责任制，持续开展道路交通、矿山、建筑施工、人员密集场所、危险化学品等领域的专项整治，坚决遏制重特大安全事故发生。进一步整顿和规范市场秩序，加强食品药品监督管理，确保人民饮食用药安全。加强物价调控，保持物价总水平基本稳定。拓展双拥共建领域，建设双拥模范城。大力发展民族团结进步事业，全面贯彻落实党的宗教工作基本方针，提升宗教事务规范化管理水平，依法管理宗教事务，坚决打击境外宗教势力渗透破坏活动，维护现有宗教格局，引导宗教与社会主义社会相适应。{CACOPAGE}

　　切实加强政府自身建设

　　艰巨的任务，重大的责任，群众的期盼，对政府工作提出了新的更高要求，我们必须加快转变政府职能，增强政府创新力、服务力、公信力、执行力，努力建设让人民满意的政府。

　　坚持解放思想，增强政府创新力。深入推进“思想大解放、树立新形象”活动和成果转化运用，做到“五个破除、五个树立”，转变发展理念，提振发展信心，鼓舞发展斗志。深入推进学习型政府建设，切实加强公务员教育管理，大力弘扬求真务实和调查研究之风，提升干部队伍素质和推动科学发展的能力。深入开展营造风清气正发展环境活动，切实改善政务环境、法制环境、人文环境、市场环境、金融环境、用人环境和企业发展环境及干事创业环境。深入推进行政体制改革和事业单位综合配套改革，进一步完善科学决策机制，形成权责一致、科学合理、务实创新、高效运行的管理体制，切实提高政府创新能力。

　　坚持执政为民，增强政府服务力。坚持以改善民生为根本，尽心竭力为群众办实事、做好事、解难事。强化公共服务，加快构建公共财政，将财力更多地投向民生、社会保障和社会事业领域，为人民群众提供更多优质公共产品和服务。强化便民服务，清理和规范行政审批事项，严格执行首问负责、限时办结、公开承诺、责任追究制度。进一步精简公文，压缩会议，提高办事效率。继续办好政府网站“政民互动”栏目，加强电子政务建设，简化办事程序，切实提高政府服务水平。

　　坚持依法行政，增强政府公信力。严格依照法定权限和程序履行职责，规范行政决策程序，提高依法行政水平。加大政务公开力度，创新政务公开方式，全面推进政务信息公开和办事公开，切实保障人民群众的知情权、参与权、监督权。自觉接受人大法律监督和政协民主监督，认真办理人大代表议案、建议和政协提案，广泛听取各民主党派、无党派人士、工商联和社会各方面的意见，支持工会、共青团、妇联、文联、残联等群众团体工作，更加重视社会舆论监督，拓宽群众监督渠道，树立政府在群众中的公信力。

　　坚持勤政廉政，增强政府执行力。认真落实改进干部作风“十项规定”、“八办要求”，强化责任意识、效率意识、创新意识，形成激情干事、拼抢干事、齐心干事的强大合力，对重点工作、重大项目和民生实事，逐项落实责任，加强督促检查，严格考核奖惩，切实提高执行力。着力推进政府系统反腐倡廉建设，坚持用制度管人、管权、管事。完善政府重大投资项目公示制和责任追究制，加强行政执法监察监督，规范工程招投标、土地出让、产权交易、政府采购、医药购销、公务消费等行为，强化领导干部任期经济责任审计。严肃查处违法违纪案件，整肃政风行风，树立政府为民、务实、清廉、高效的良好形象。

　　各位代表，2012年的目标宏伟而艰巨，困难和挑战考验着我们，机遇和期待召唤着我们，责任和使命激励着我们。让我们在自治区党委、政府和市委的坚强领导下，高举中国特色社会主义伟大旗帜，深入实践科学发展观，以更加开放的思想观念、更加执著的奋斗精神、更加扎实的工作作风，务实苦干，跨越发展，强市富民，为建设新固原而努力奋斗!
